# Single-cell chromatin accessibility landscape of human umbilical cord blood in trisomy 18 syndrome

**DOI:** 10.1186/s40246-021-00338-z

**Published:** 2021-06-30

**Authors:** Xiaofen Qiu, Haiyan Yu, Hongwei Wu, Zhiyang Hu, Jun Zhou, Hua Lin, Wen Xue, Wanxia Cai, Jiejing Chen, Qiang Yan, Weier Dai, Ming Yang, Donge Tang, Yong Dai

**Affiliations:** 1 Department of Clinical Medical Research Center, Guangdong Provincial Engineering Research Center of Autoimmune Disease Precision Medicine, The First Affiliated Hospital of Southern University of Science and Technology, The Second Clinical Medical College of Jinan University, Shenzhen People’s Hospital, Shenzhen, Guangdong 518020 People’s Republic of China; 2 Guangxi Key Laboratory of Metabolic Diseases Research, Department of Clinical Laboratory of Guilin, No. 924 Hospital, 541002 Guilin, Guangxi People’s Republic of China; 3 College of Life Science, Guangxi Normal University, Guilin, Guangxi 541004 People’s Republic of China; 4 College of Natural Science, University of Texas at Austin, Austin, TX 78712 USA

**Keywords:** Trisomy 18 syndrome, single-cell sequencing, Transcription factors, Aneuploidy, Developmental regulation

## Abstract

**Background:**

Trisomy 18 syndrome (Edwards syndrome, ES) is a type of aneuploidy caused by the presence of an extra chromosome 18. Aneuploidy is the leading cause of early pregnancy loss, intellectual disability, and multiple congenital anomalies. The research of trisomy 18 is progressing slowly, and the molecular characteristics of the disease mechanism and phenotype are still largely unclear.

**Results:**

In this study, we used the commercial Chromium platform (10× Genomics) to perform sc-ATAC-seq to measure chromatin accessibility in 11,611 single umbilical cord blood cells derived from one trisomy 18 syndrome patient and one healthy donor. We obtained 13 distinct major clusters of cells and identified them as 6 human umbilical cord blood mononuclear cell types using analysis tool. Compared with the NC group, the ES group had a lower ratio of T cells to NK cells, the ratio of monocytes/DC cell population did not change significantly, and the ratio of B cell nuclear progenitor and megakaryocyte erythroid cells was higher. The differential genes of ME-0 are enriched in Human T cell leukemia virus 1 infection pathway, and the differential peak genes of ME-1 are enriched in apopotosis pathway. We found that CCNB2 and MCM3 may be vital to the development of trisomy 18. CCNB2 and MCM3, which have been reported to be essential components of the cell cycle and chromatin.

**Conclusions:**

We have identified 6 cell populations in cord blood. Disorder in megakaryocyte erythroid cells implicates trisomy 18 in perturbing fetal hematopoiesis. We identified a pathway in which the master differential regulatory pathway in the ME-0 cell population involves human T cell leukemia virus 1 infection, a pathway that is dysregulated in patients with trisomy 18 and which may increase the risk of leukemia in patients with trisomy 18. CCNB2 and MCM3 in progenitor may be vital to the development of trisomy 18. CCNB2 and MCM3, which have been reported to be essential components of the cell cycle and chromatin, may be related to chromosomal abnormalities in trisomy 18.

**Supplementary Information:**

The online version contains supplementary material available at 10.1186/s40246-021-00338-z.

## Introduction

Trisomy 18 syndrome is a type of common aneuploidy caused by the presence of an extra full chromosome 18 trisomy, mosaic trisomy, or partial trisomy 18 [1]. Edwards et al. described the first reported infants in 1960, and it is also known as Edwards syndrome (ES) [2, 3]. ES is the second most common autosomal trisomy syndrome with a frequency of around 1 in 6000 live births after trisomy 21 (known as down syndrome) [4]. Patients with trisomy 21 can survive as chromosome 21 is the minimal number of chromosomes transcribed in humans, but trisomy 18 is viable after birth but die within a few months of birth [5]. The main clinical features include prenatal growth deficiency, characteristic craniofacial features, craniofacial features, distinctive hand posture of overriding fingers, nail hypoplasia, short hallux, and major malformations (particularly involving the heart) [6]. It is generally believed that the extra chromosome exists because of non-disjunction. In parental origin analysis, additional chromosomes are usually of maternal origin as a result of chromosomal segregation erroneous in meiosis or post-zygotic mitosis [7, 8]. A study has shown changes in gene expression in trisomy 18 syndrome. The British team of David R. Fitz Patrick, based on cDNA array analysis of trisomy syndromes, found that three-body on chromosome gene transcription, on average, increased only 1.1 times. They detected the expression level differences between chromosomes, suggesting that the genomic regulation mechanisms may act at greater distances than ever thought; that is, a chromosome increase may affect other chromosomes gene transcription levels. Their data support a model, in which trisomy leads to a slightly raise of chromosome gene that leads to secondary transcription, universality, and more extreme imbalance, and the extent of this faulty regulation may determine the severity of the phenotype [9]. To date, we know little about the genomic regulatory of trisomy 18 syndrome. Human aneuploidy is the major cause of early pregnancy loss, intellectual disability, and multiple congenital anomalies, and trisomy is the most common aneuploidy in humans [10]. Previous studies focused mostly on trisomy 21 syndrome; however, the pathogenic mechanisms of trisomy 18 syndrome remain largely unknown due to the high mortality associated with aneuploidy [11]. There is an urgent need to understand the mechanisms of gene expression regulation in trisomy 18.

Traditional high-throughput sequencing strategies that use intact tissue or cells are limited to averaging the constituent cell profiles. Thanks to great advancements in unbiased single-cell genomic assays, a single-cell assay for transposase-accessible chromatin using sequencing (sc-ATAC-seq) has been developed [12]. It leverages the concept that every single cell can only occupy a single position in the landscape of cell types. This assay can reveal several layers of gene regulation in a single assay, including genome-wide identification of cis-elements, inference with TF binding and activity, and nucleosome positions [13, 14]. Sc-ATAC-seq not only merely adapted to disentangling cell type heterogeneity, but it also captures the chromatin regulatory landscape that governs transcription in each cell type in complex tissues. To characterize the cellular and molecular changes associated with trisomy 18 syndrome in greater detail, and to gain further insights into the mechanisms influencing one trisomy 18 syndrome traits, we subjected human umbilical cord blood of the trisomy 18 syndrome patient and one control to a single-cell assay for transposase-accessible chromatin using sequencing (sc-ATAC-seq). The latest-generation DNA sequencing technology, single-cell assay for transposase-accessible chromatin (sc-ATAC-seq) is expected to eventually reveal the mechanisms of gene expression regulation and the related specific molecular markers in cell clusters.

In this approach, we applied a commercial system (10× Genomics) to perform sc-ATAC-seq on nanolite-scale droplets, which can generate high-quality single-cell chromatin accessibility profiles on a large scale. We performed sc-ATAC-seq to measure chromatin accessibility in 11,611 single cells derived from 2 samples, one trisomy 18 syndrome patient and one healthy donor. Using the sc-ATAC-seq data, we obtained 11 distinct major clusters of cells, identified as 6 human umbilical cord blood mononuclear cell types using single-cell ATAC-seq analysis tool [15] and characterized the driving regulatory factors and differentially accessible loci that defined each cluster. We set out to generate a single-cell atlas of chromatin accessibility from healthy and the trisomy syndrome human umbilical cord blood samples. Finally, cell-type-specific gene regulatory network analysis of these differential accessibility-related loci genes was carried out at single-cell resolution, and disease-related genes were predicted. These screened disease-related transcription factors (TFs) and their target genes provide a basis for further research that will improve our understanding of trisomy 18 syndrome.

## Method

### Study group

The criteria for inclusion in the disease group for this study were G banding karyotype was 47, XY, +18 according to the international system for human cytogenetic nomenclature(ISCN), 2016. One umbilical cord blood (UCB) sample was collected with informed consent from a 32-year-old, pregnant, healthy donor as a natural control (NC). The G banding karyotype of the healthy control woman’s fetus was 46, XY. Patients UCB samples recruited for this study were collected with informed consent from a 20-year-old pregnant donor as the study subject (trisomy 18 syndrome, also known as Edwards syndrome, ES). The G banding karyotype of this donor’s fetus was 47, XY, +18. Each fetus was at a gestational age of 18-22 weeks. Samples were stored in ethylene diamine tetraacetic acid (EDTA) anticoagulant tubes and transported to the laboratory within 1 h. Cord blood mononuclear cells (CBMCs) were isolated by Ficoll-Hypaque density gradient centrifugation according to standard protocols.

### Sc-ATAC-seq using the 10× Chromium platform

All protocols of this study to generate scATAC-seq data on the 10× Chromium platform, including nucleus isolation and transposition, library construction, and instrument and sequencing parameters, are described below and available here: https://support.10xgenomics.com/single-cell-atac.

#### Nuclei isolation and transposition

The CBMC thawing, isolation, washing, and counting of nuclear suspensions were performed according to the Demonstrated Protocol: Nuclei Isolation for Single Cell ATAC Sequencing (10× Genomics). Step 1: CBMCs were gently thawed in a water bath at 37 °C for 1–2 min and resuspended in PBS + 0.04% BSA. Step 2: 100,000–1,000,000 cells were added to a 2-ml microcentrifuge tube and centrifuged at 300 rcf for 5 min at 4 °C. All supernatant was removed without disrupting the cell pellet. Step 3: add 100 μl of chilled lysis buffer (10 mM Tris-HCl (pH 7.4), 10 mM NaCl, 3 mM MgCl2, 0.1% Tween-20, 0.1% Nonidet P40 Substitute, 0.01% digitonin and 1% BSA) was added. The sample was incubated for 3 min on ice. Step 4: add 1 ml chilled wash buffer (10 mM Tris-HCl (pH 7.4), 10 mM NaCl, 3 mM MgCl2, 0.1% Tween-20 and 1% BSA) was added to the lysed cells, which were mixed with a pipette 5× and centrifuged at 500 rcf for 5 min at 4 °C. The supernatant was removed without disrupting the nuclear pellet. Based on the nuclear concentration measured before step 2 and assuming ~ 50% nuclear loss during cell lysis, the nuclear pellet was resuspended in chilled diluted Nuclei Buffer (10× Genomics, PN-2000153). The cell suspension was passed through a 40-μm Flowmi Cell Strainer, and the cell concentration was determined using a Countess II FL Automated Cell Counter or a heamocytometer. We proceeded immediately to the Chromium Single Cell ATAC Reagent Kits protocol (CG000168). Nuclear suspensions were incubated in a transposition mix that included a transposase. The transposase entered the nuclei and preferentially fragmented the DNA in regions of the accessible chromatin while adapter sequences were added to the ends of the DNA fragments.

#### Sc-ATAC-seq library construction

Sc-ATAC-seq libraries were prepared according to the Chromium Single Cell ATAC Reagent Kits User Guide (10× Genomics; CG000168 Rev B). Briefly, nuclei were transposed in a bulk solution; then, using a microfluidic chip, the nuclei were partitioned into nanolitre-scale gel beads-in-emulsion (GEMs). GemCode Technology sampled a pool of ~ 750,000 10× barcodes to separately and uniquely index the transposed DNA of each individual cell. GEMs were generated by combining barcoded gel beads, transposed nuclei, a master mix, and partitioning oil on a Chromium Chip E. The nuclei were delivered at limiting dilution to achieve single-nucleus resolution; therefore, the majority (~ 90–99%) of generated GEMs contained no nuclei, while the remainder largely contained a single nucleus. The gel bead was dissolved after GEM generation. Oligonucleotides containing an Illumina® P5 sequence, a 10× barcode and a Read 1 (Read 1N) sequence were released and mixed with DNA fragments and master mix. The thermal cycling of the GEMs produced 10×-barcoded single-stranded DNA. After incubation, the GEMs were broken, and pooled fractions were recovered. Next, silane magnetic beads were used to remove leftover biochemical reagents from the post-GEM reaction mixture. Solid-phase reversible immobilization (SPRI) beads were used to eliminate unused barcodes from the sample. In the end, the sample index P7 and Read 2 (Read 2N) sequences were added during library construction via PCR. The final scATAC-seq libraries contained the P5 and P7 primers used in Illumina® bridge amplification. Libraries were generated and sequenced, and 10× barcodes were used to associate individual reads with the individual partitions and thereby to each cell.

#### Data quality control and genome alignment

To improving data quality, fix the occasional sequencing error in barcodes, the fragments get associated with the original barcodes. The barcode sequence is first obtained from the “I2” index reads. Cell Ranger ATAC(https://support.10xgenomics.com/single-cell-atac/software/overview/welcome) performs reference-based analysis and requires adapter and primer oligo sequence to be trimmed off before mapping confidently. The cut adapt [16] tool was used to identify the primer sequence at the end of each read and trim it from the read before alignment. Then, the trimmed read-pairs are aligned to a specified reference using BWA-MEM [17] with GRCh38. Reads less than 25 bp are not aligned by BWA-MEM. These unaligned reads are marked as unmapped. Due to PCR amplification, a barcode fragment is sequenced multiple times. Duplicate reads are obtained by identifying a set of read pairs that have the same mapping position on the reference genome at the 5′ end of all barcodes, where R1 and R2 have the same mapping position on the reference genome. Unique read pairs were reported as a fragment in the fragment file.

#### Dimensionality reduction and cluster differential accessibility

We used Signac v0.2.5 (https://github.com/timoast/signac) and Seurat [18] performs dimensionality reduction and cluster differential accessibility. Notably, the method used for data analysis is developing fast, and we used a currently widely applied method to re-analyze our data compared to our previous paper [19]. Since the data under the single-cell resolution is sparse, we first perform dimensionality reduction to cast it into a lower-dimensional space, which has the advantage of de-noising. We performed a graph-based clustering and visualization via UMAP [20]. The data normalized to the unit norm before performing graph-based clustering and UMAP projection.

#### Marker gene identification and cell-specific-type annotation

Marker gene identification and cell-specific-type annotation were performed according to the Cell Type Annotation Strategies 2 for Single Cell ATAC-Seq Data (10× Genomics; CG000234 Rev A), the strategy of cell type annotation employs gene activation scores of cell type markers with known In this study, cell type-specific cut site distribution was exported from Loupe Cell Browser (10× Genomics,) by loading the fragments.tsv.gz to peak viewer and exporting cut sites per cell type per window. Cell-type-specific peaks were defined as the top 200 enriched peaks of the selected cell type over all other cell types. Background was defined as 500 sets of 200 randomly selected peaks. The cell type generating the maximum enrichment score was annotated to the cell. Cell clusters were annotated using canonical markers of known cell types. The obtained cell cluster and the cellular marker gene that has been reported and included on the cell marker website were mapped (http://biocc.hrbmu.edu.cn/CellMarker/index.jsp).

#### Peak-related genes annotation

The detected peaks are open chromatin-rich regions with potential regulatory functions, and looking at the location of peaks and genes, it can provide insight into chromatin accessibility characteristics. We used BEDTools [21] to map each peak to a gene level based on the closest transcription start site within 1000 bases upstream or 100 bases downstream of the TSS.

#### Peak-related genes GO enrichment and genes pathway enrichment analysis

We use Gene Ontology (GO) to analyze peak-related Gene. Firstly, all peak-related genes were mapped to GO terms in the Gene Ontology database (http://www.geneontology.org/), gene numbers were calculated for every term, significantly enriched GO terms in peak-related genes comparing to the genome background were defined by hypergeometric test. *P* value < 0.05 is considered to be significantly enriched term. The calculating formula of *P* value is

$$ P=\sum \limits_{i=0}^{m-1}\frac{\left(\begin{array}{c}M\\ {}i\end{array}\right)\kern0.5em \left(\begin{array}{c}N-M\\ {}n-i\end{array}\right)}{\left(\begin{array}{c}N\\ {}i\end{array}\right)} $$

Here, N is the number of all genes with GO annotation; n is the number of peak-related genes in N; M is the number of all genes that are annotated to the certain GO terms; m is the number of peak-related genes in M. The calculated p values were gone through FDR correction, taking FDR ≤ 0.05 as a threshold. GO terms meeting this condition were defined as significantly enriched GO terms in peak-related genes. This analysis was able to recognize the main biological functions that peak-related genes exercise.

We use the Kyoto Encyclopedia of Genes and Genomes (KEGG) to make the peak-related genes pathway enrichment analysis. KEGG is the major public pathway-related database. Pathway enrichment analysis identified significantly enriched metabolic pathways or signal transduction pathways in peak-related genes compared with the whole genome background. The calculating formula is the same as that in the GO analysis.

$$ P=\sum \limits_{i=0}^{m-1}\frac{\left(\begin{array}{c}M\\ {}i\end{array}\right)\kern0.5em \left(\begin{array}{c}N-M\\ {}n-i\end{array}\right)}{\left(\begin{array}{c}N\\ {}i\end{array}\right)} $$

Here, N is the number of all transcripts that with KEGG annotation, n is the number of peak-related genes in N, M is the number of all transcripts annotated to specific pathways, and m is number of peak-related genes in M. The calculated p value was gone through FDR correction, taking FDR < 0.05 as a threshold. Pathways meeting this condition were defined as significantly enriched pathways in peak-related genes.

#### TF motif identification and enrichment analysis

The peaks obtained are usually enriched in transcription factor (TF) binding sites, and the presence of certain groups can predict the activity of transcription factors. We use the MOODS (https://github.com/jhkorhonen/MOODS). The Python library packaged in Cell Ranger ATAC was used to scan each peak for matches to the group positions of transcription factors in the JASPAR database.

## Result

### Quality control of the scATAC-seq profile

**Fig. 1:**
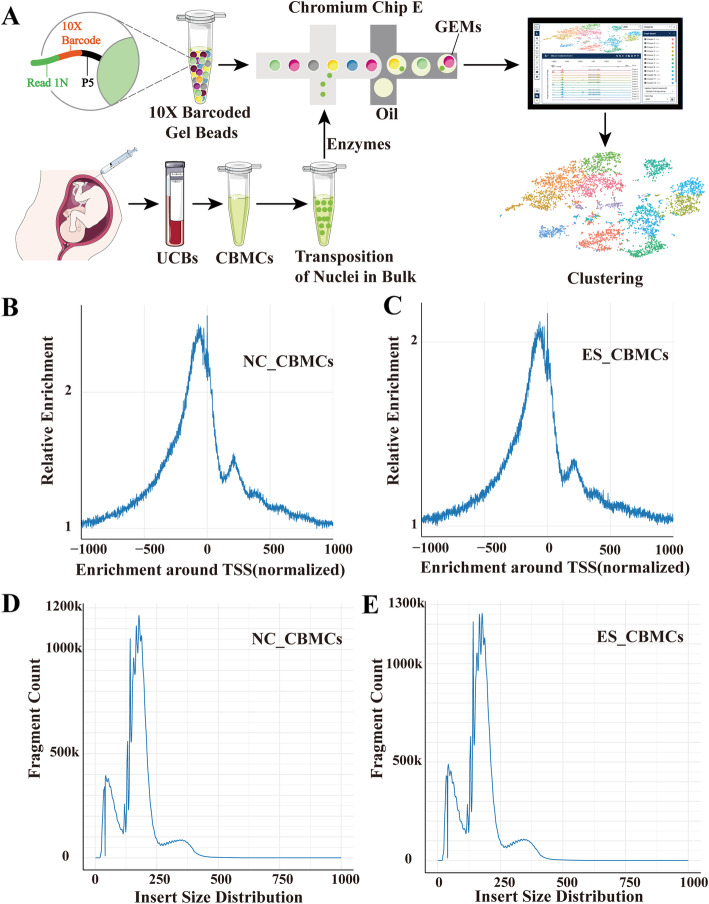
Quality control of the scATAC-seq profile. A Schematic of the single-cell assay for transposase-accessible chromatin using sequencing of UBMC nuclei isolated and transposition. **B, C** The signal distribution map around TTS after normalization of NC_UBMCs and ES_UBMCs. The horizontal coordinate is the position relative to the TSS, and the vertical coordinate is the relative signal strength. **D, E** Length distribution of NC_UBMCs and ES_UBMCs library inserts for each sample. The horizontal axis indicates the length of the inserts, and the vertical axis indicates the proportion of inserts of that length to the total number of inserts

We used the commercial system Chromium platform (10× Genomics) to perform scATAC-seq in nanoliter-sized droplets to measure single-cell chromatin accessibility landscape of umbilical cord blood [22, 23]. Briefly, we disaggregated two cord blood mononuclear cells from the healthy control (NC) and the trisomy 18 syndrome (Edwards syndrome, ES) patient, and nuclei were isolated from each single-cell suspension and transposed in bulk with the transposase Tn5 (Fig. 1A). After removing additional cellular debris and minimizing mitochondrial contamination, a total of 11,611 cord blood mononuclear cells (6,315 cells of NC; 5,296 cells of ES) passing the filter yielded 5689 median fragments, a 9.5% fraction of transposition events in peaks in the cell barcodes, and a 44.6% fraction of fragments overlapping the targeted region. The median number of unique fragments per cell barcode in the NC_CBMC library after normalization was 226,088,665, and that in the ES_CBMC library was 170,248,377. More than 2-fold enrichment of fragments proximal to TSSs (relative to distal regions) from the NC_CBMC data and ES_CBMC data was observed. These data reflect a high fraction of fragments captured within open rather than closed chromatin (Fig. 1B, C). Since the Tn5 transposase preferentially attacks open chromatin regions, most DNAs were generally short fragments containing no or only one nucleosome, while the long fragments of NC_CBMC data and ES_CBMC data containing multiple nucleosomes exhibited a distinct fragment distribution in terms of content distribution. The library insertion size distribution for each sample was calculated using the comparison information from both ends of the reads (Fig. 1D, E). On average, at 27.8 × 103 unique fragments mapped to the nuclear genome, approximately 38.1% of Tn5 insertions were within peaks present in aggregated profiles from all cells, a proportion comparable to published acceptable ATAC-seq profiles.

### Single-cell chromatin accessibility profile of umbilical cord blood from major immune cell types

**Fig. 2:**
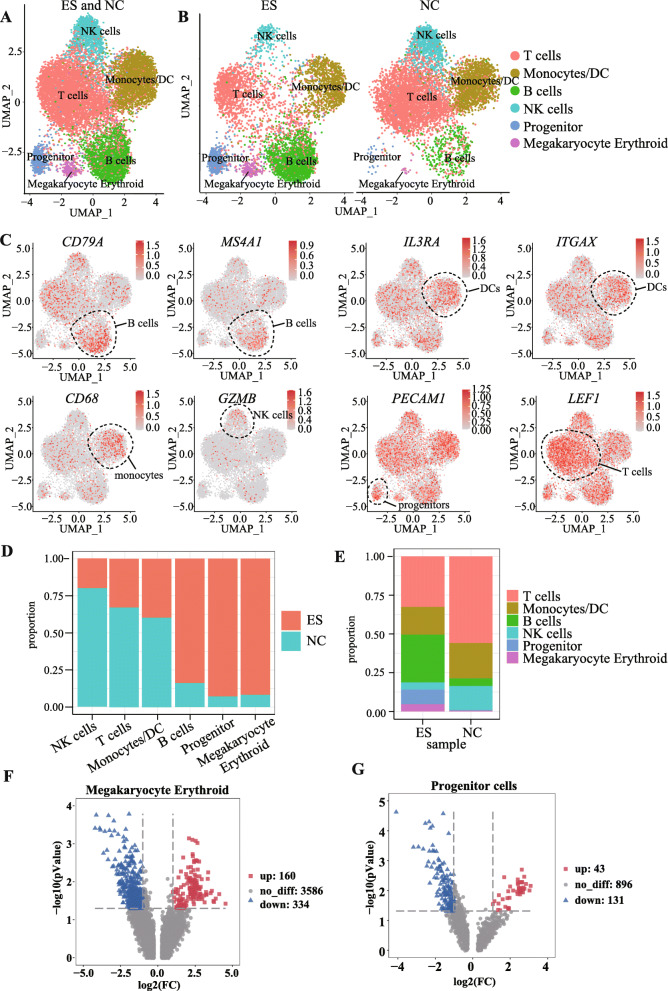
Landscape of trisomy 18 and control umbilical cord blood single nucleated cells. **A** UMAP of ES_Library and NC_Library. **B** UMAP of ES_Library and UMAP of NC_Library. **C** Cell-specific marker expression of clusters. **D** Cell ratio of ES_Library and NC_Library in sample. **E** Cell ratio of ES_Library and NC_Library in cell type. **F** Volcano plot of megakaryocyte erythroid and progenitor cells

The sc-ATAC-seq reads from 11,611 cells from both NC and ES use Cell Ranger ATAC and UMAP cluster to perform dimensionality reduction clustering and identified a total of 13 cell clusters. The obtained cell cluster and the cellular marker gene that has been reported and included on the cell marker website (http://biocc.hrbmu.edu.cn/CellMarker/index.jsp) were mapped. We assigned the 13 cell clusters (Supplementary Figure S1A, and Supplementary Figure S1B) identified as the 6 major immune cell types known to be found in the umbilical cord blood: T cells, B cells, monocytes/dendritic cell (DC), natural killer cells (NK), progenitor cells, and megakaryocyte erythroid (ME) (Fig. 2A, analysis of total 13 cell clusters identified 6 distinct populations of cells which are demonstrated accessibility of open chromatin regions linked to cell-specific genes: B cells were assigned by *MS4A1*, *CD79A*, and *CD79B* gene promoter cell-specific genes) [24]. Cell populations expressing ITGAX, CD36, and IL3R1 were identified as monocytes/dendritic cells (DC) [25, 26]. Natural killer (NK) cells were identified by *GZMB* and *NKG7* gene promoter cell-specific genes. T cells were identified by *LEF1* gene promoter cell-specific genes [27], and megakaryocyte erythroid (ME) cells were identified by *KIT*, *CD36*, *CD38* (https://www.bio-rad-antibodies.com/human-immune-cell-markers-selection-tool.html#cell=megakaryocyte-erythroid-cells) gene promoter cell-specific genes [28]. Progenitor cells were identified by *PECAM1* cell-specific genes [29] (Fig. 2C). After determining the 6 cell types that belonged to the seven cell clusters of cord blood mononuclear cells, we evaluated cell abundances and calculated the composition ratio of each group of cells. Among the six major cell clusters, three were significantly larger in trisomy 18 syndrome. The ratio of B cells, progenitor cells, and megakaryocyte erythroid cells were significantly higher in the ES than the NC group. The ratios of natural killer (NK) cells, T cells, and monocyte/dendritic cell (ES) cells were significantly lower in the ES group than in the NC group (Fig. 2D). We next counted the cellular composition of the ES and NC groups (Fig. 2E). Our results suggest that certain peak genes in progenitor cells, and megakaryocyte erythroid (ME) cells play an essential role in the development of the ES phenotype. To determine the differences in the number of differentially accessible peak regions between the six classes of identified cell populations, we performed volcano mapping analysis. Peaks with *P <* 0.05, |log_2_FC| > 1 is considered a significant difference. The number of differential peaks in T cell, B cell, monocyte/DC, and NK cell populations are 0, 1, 2, 3, respectively. Compared to controls (NC), a total of 494 differentially accessibility chromatin were obtained in the ME cell populations of trisomy 18 (ES), of which 160 peak (p < 0.05, log_2_FC > 1) and 334 peak expression (p < 0.05, log_2_FC < −1) (Fig. 2F). After annotating 494 difference peaks, 483 genes were obtained. A total of 174 significantly different peaks were obtained from the progenitor cells, of which 43 indicated upregulation (p < 0.05, log_2_FC > 1) and 131 peaks downregulation (p < 0.05, log_2_FC < − 1) (Fig. 2G). After annotating 174 difference peaks, 173 genes were obtained. Next, we will conduct a more detailed analysis of the progenitor cells and megakaryocyte erythroid (ME) cells that have obtained differential peaks. This result pointed us in the direction of our next analysis by implying that ME and progenitor cell populations play important roles in the development of trisomy 18, followed by a further analysis of the heterogeneity of ME cell populations and P cells in the control and disease subjects.

### Human T cell leukemia virus 1 infection pathway disorder in megakaryocyte erythroid cells

**Fig. 3:**
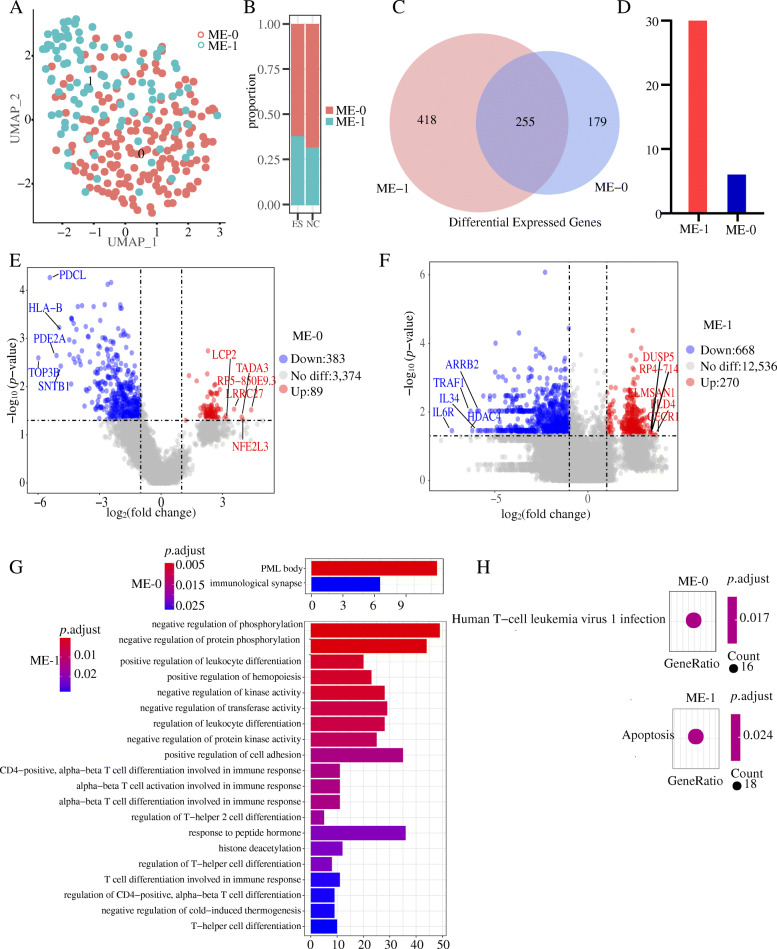
ME cell population clustering analysis with differentially accessible peak analysis. **A** UMAP plot of megakaryocyte erythroid cells. Cluster 0 is marked 0, and cluster 1 is marked 1. **B** Proportions of cells in the ES and NC groups: megakaryocyte erythroid cells, cluster 0 labelled ME-0, cluster 1 labelled ME-1. **C** Genes co-regulated by ME-0 and ME-1 had significantly differential peaks (p < 0.05, log_2_FC > 1). **D** Number of motifs significantly upregulated by ME-0 and ME-1 (p < 0.05, log_2_FC > 0.58). **E** ME-0 differentially expressed genes (p < 0.05, |log_2_FC| > 1). Blue dots indicate downregulated genes, red dots indicate upregulated genes, and grey dots indicate genes that are satisfactorily significantly different and label the five genes with the largest multiplicity of differences. **F** ME-1 differentially expressed genes (p < 0.05, |log_2_FC| < 1). **G** Results of GO enrichment analysis of ME-0 and ME-1. **F** Results of KEGG enrichment analysis of ME-0 and ME-1

We identified a total of 267 megakaryocyte erythroid (ME) cells. The proportion of megakaryocyte erythroid (ME) cells in ES and NC was 0.461(244/5296) and 0.035(22/6315), respectively. We performed a further dimension reduction analysis and UMAP (uniform manifold approximation and projection) of the ME cell population (Fig. 3A). The ratio of ME0 to ME1 is not significantly different between the ES group and the NC group(Fig. 3B). Among the differentially expressed peak genes, there are 255 peak genes shared by ME0 and ME1 cells, 179 peak genes specific to the ME0 cell group, and 418 unique peak genes for ME1 (Fig. 3C). There are 7 and 30 different motifs for ME-0 and ME-1 respectively (P < 0.05, FC > 1.2) (Fig. 3D). To obtain the number of regions of differentially accessible peaks gene for ME-0 and ME-1, we performed a volcano diagram analysis, and we obtained a total of 472 significant different peak genes (89 upregulated, 383 downregulated, p < 0.05, |log_2_FC| > 1) for ME-0 and 938 significant different peak genes (270 upregulated, 668 downregulated, p < 0.05, |log_2_FC| > 1) for ME-1 (Fig. 3E, F). To identify the differential functions and related pathways of the two populations of ME cells, we performed GO and KEGG analysis of the peak genes of the significantly different peaks (p-adjust < 0.05, |log_2_FC| > 1). The GO analysis showed that the differentially expressed genes of ME cells were associated with biological functions such as negative regulation of phosphorylation and negative regulation of protein phosphorylation (Fig. 3G). The differential genes of ME-0 are enriched in Human T cell leukemia virus 1 infection pathway, and the differential peak genes of ME-1 are enriched in apopotosis pathway.

### Downregulation of MCM3 and CCNB2 in progenitor cells

**Fig. 4:**
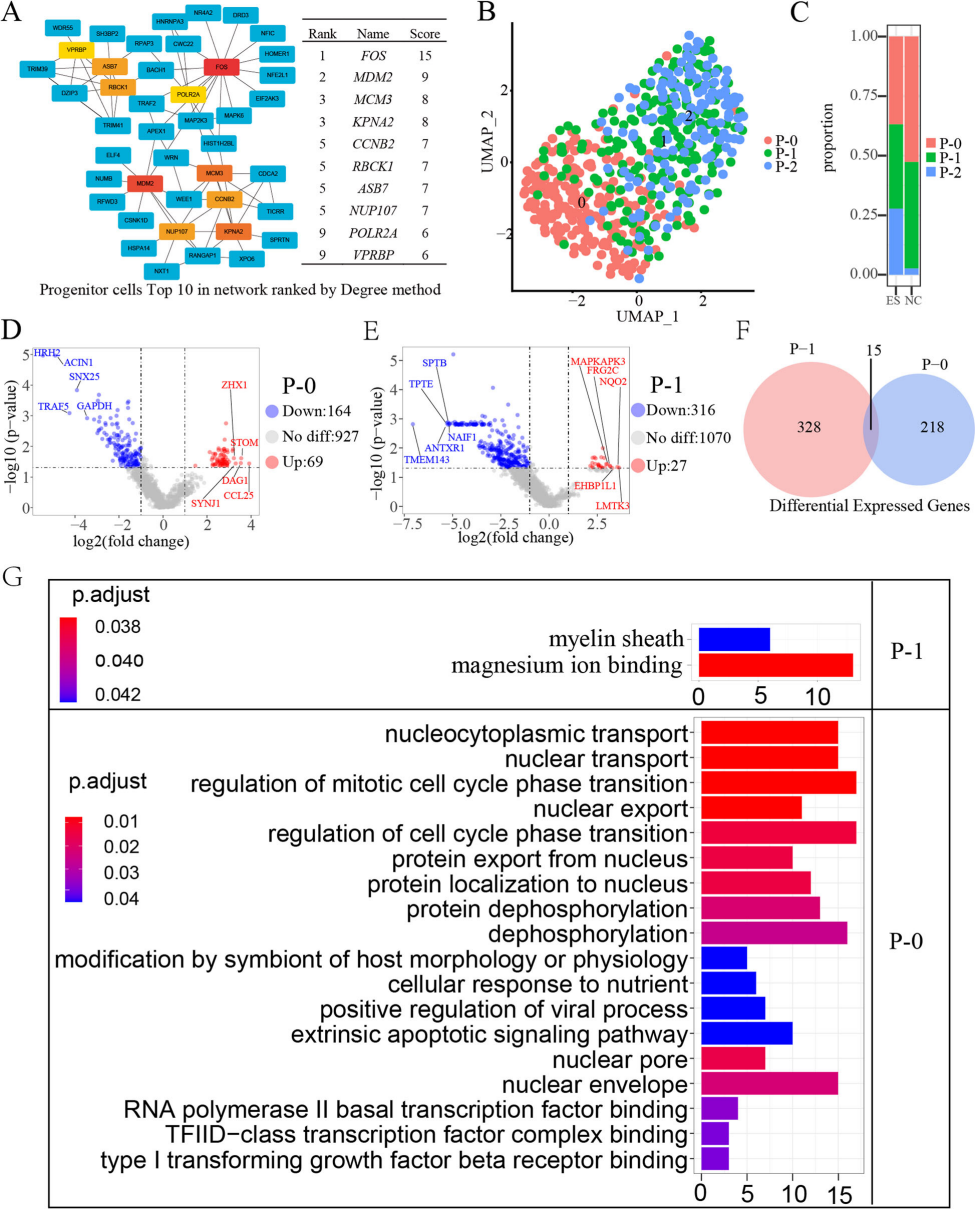
Molecular characterization of progenitor cells. A Top 10 network progenitor cells ranked by the degree method. The network diagram shows how the hub gene interacts with other genes. The redder the box, the higher the score, indicating a more critical gene. **B** UMAP plot of progenitor cells. Cluster 0 is marked 0, and cluster 1 is marked 1. **C** Proportion of cells in the ES and NC groups in progenitor cells, cluster 0 labelled P-0, cluster 1 labelled P-1. **D** P-0 differentially expressed genes (p < 0.05, |log2FC| > 1). Blue dots indicate downregulated genes, red dots indicate upregulated genes, and grey dots indicate genes that are satisfactorily significantly different and label the five genes with the largest multiplicity of differences. **E** P-1 differentially expressed genes (p < 0.05, |log2FC| < 1). **F** Genes co-regulated by P-0 and P-1 had significantly different peaks (p < 0.05, log_2_FC > 1). **G** Results of GO enrichment analysis of P-0 and P-1

Cord blood contains a large number of stem cells, including hematopoietic stem cells and a variety of other stem cells, collectively known as cord blood stem cells/progenitor cells. We captured a total of 538 progenitor cells, including 500 in the ES group and 38 in the NC group (Fig. 1B, C). After differential analysis, 1070 peak genes were obtained, among which 43 peaks were significantly upregulated and 131 peaks were significantly downregulated (p < 0.05, log_2_FC > 1), and there was no significant difference for 896 peaks (Fig. 2G). To assess key genes for Chromatin accessibility regions of the P cell population, we used the cytoHubba program of Cytoscape (3.8.0) to calculate the hub genes (top 10, MCC/degree) (Fig. 4A). The 10 hub genes we got are FOS, MDM2,MCM3, KPNA2, CCNB2, RBCK1, ASB7, NUP107, POLR2A, VPRBP. To further investigate the molecular characteristics of the trisomy 18 progenitor cells, we performed a UMAP (uniform manifold approximation and projection) analysis of the progenitor cells (Fig. 4B). Compared with the NC group, the proportion of p-0 cells in the ES group decreased, the proportion of P-1 cell populations decreased, and the proportion of P-2 cell populations increased (Fig. 4C). To obtain genes differentially expressed in trisomy 18, we performed volcano mapping analysis of three cell populations of P cells, and we obtained the P-0 and P-1 differentially expressed genes (P < 0.05, log_2_FC > 1), but no significant differences for P-2. Compared with the trisomy 18 control, the trisomy 18 samples had 233 significantly different peak genes in P-0, including 164 downregulated peak genes (P < 0.05, log_2_FC < − 1) and 69 upregulated peak genes (P < 0.05, log_2_FC > 1) (Fig. 4D), and 343 significantly different peak genes in P-1, including 27 downregulated genes (P < 0.05, log_2_FC < − 1) and 336 upregulated peak genes 316 genes (P < 0.05, log_2_FC > 1) (Fig. 4E). There are only 15 differential peak genes in both P-0 and P-1 cell populations, indicating that P-0 and P-1 are two different progenitor cell populations (Fig. 4F). To determine the main biological functions performed by P-0 and P-1 differentially expressed genes, we performed a GO enrichment analysis of the differentially expressed peak genes (Fig. 4F). We noticed that the differential peak genes of the P-0 cell population are enriched in the regulation of mitotic cell cycle phase transition, regulation of cell cycle phase transition, RNA polymerase II basal transcription factor binding and other pathways.

## Discussion

Here, we set out to generate a single-cell atlas of umbilical cord blood mononuclear cell chromatin accessibility. We applied sc-ATAC-seq to measure chromatin accessibility in 11,611 single cells derived from 2 samples, representing 6 major immune cell types: T cells, monocytes/DC, B cells, NK cells, progenitor, megakaryocyte erythroid. Compared with the NC group, the ES group had a lower ratio of T cells to NK cells, the ratio of monocytes/DC cell population did not change significantly, and the ratio of B cell nuclear progenitor and megakaryocyte erythroid cells was higher. This result suggests that the immune system of trisomy 18 may have changed. In 1994, G. Makrydimas et al. [30] enumerated lymphocyte subpopulations in fetal blood obtained by cordocentesis from eight trisomy 18 fetuses at 20–36 weeks’ gestation. Compared with values in chromosomally normal fetuses, in trisomy 18, the mean T and natural killer (NK) cell counts were significantly lower, while the mean B cell count was not significantly different. Here, the number of B cells was significantly increased in the disease group in our single-cell analysis. These findings demonstrate that in trisomy 18, there is abnormal intrauterine development of the immune system.

Compared with those from the healthy donor (NC), a total of 494 peak genes in megakaryocyte erythroid cells were differentially expressed in the trisomy 18 syndrome (ES) donor, and a total of 174 peak genes in progenitor cells were differentially expressed in the trisomy 18 syndrome (ES) donor. The number of differential peaks in T cell, B cell, monocyte/DC, and NK cell populations are 0, 1, 2, 3, respectively. The results suggest that progenitor cells and megakaryocyte erythroid cells may play a more important role in the occurrence and development of trisomy 18 than other cell populations.

We identified a pathway in which the master differential regulatory pathway in the ME-0 cell population involves human T cell leukemia virus 1 infection, a pathway that is dysregulated in patients with trisomy 18 and which may increase the risk of leukemia in patients with trisomy 18. Our experimental results show the proportion of megakaryocyte erythroid (ME) cells in ES and NC was 0.461(244/5296) and 0.035(22/6315), respectively. Similarly, Anindita Roy et al. [31] showed that the 40-fold increase in childhood megakaryocyte-erythroid and Down syndrome implicates trisomy 21 (T21) in perturbing fetal hematopoiesis. Due to the increased number of ME cell populations in trisomy 18, we were motivated to dig deeper into the transcription factors in ME cell populations that would cause differences in gene expression. Human T cell leukemia virus 1 infection causes serious human diseases, such as leukemia, which describes the tumor features of trisomy 18 syndrome [32, 33]. Our results suggest that transcriptional dysregulation contributes to the development and progression of the trisomy 18 phenotype, that additional chromosomes may contribute to the increase in ME cell populations, and that genes of the human T cell leukemia virus 1 infection pathway are dysregulated, but the mechanism of transcriptional deregulation is unclear. Further clarification is needed.

The biological function of differentially expressed genes in P cell populations, those with a higher degree of accessibility to chromatin regions, was associated with cell cycle regulation and chromatin stability. Among them, Cyclin B2 (CCNB2) is a member of the cyclin family and is an essential component of the cell cycle regulatory machinery. Cyclin B2 may play a key role in transforming growth factor beta-mediated cell cycle control [34]. The downregulation of CCNB2 may lead to chromosome segregation abnormalities in trisomy 18. MCM3 (minichromosome maintenance complex component 3) is a highly conserved mini-chromosome maintenance protein (MCM) that is involved in the initiation of eukaryotic genome replication [35]. The hexameric protein complex formed by MCM proteins is a key component of the prereplication complex and may be involved in the formation of replication forks and the recruitment of other DNA replication-related proteins. Diseases associated with MCM3 include grade III astrocytoma and lung cancer. Among its related pathways are the regulation of activated PAK-2p34 by proteasome-mediated degradation and the E2F transcription factor network. The results showed that these differentially expressed genes primarily regulated the cell cycle phase transition, mitotic cell cycle phase transition, and nucleocytoplasmic transport. It has been shown that compared to normal diploid cells, G2 may be approximately 3 times longer in trisomy cells than in normal cells [36]. Our results indicate that the genes that control the mitotic cycle in trisomy 18 have altered chromatin accessibility, and our analysis yielded key genes related to the cell cycle: CCNB2 and MCM3, which have been reported to be essential components of the cell cycle regulatory machinery, and the latter is one of the highly conserved mini-chromosome maintenance proteins. We found that CCNB2 and MCM3 may be vital to the development of trisomy 18. CCNB2 and MCM3, which have been reported to be essential components of the cell cycle and chromatin, it may be related to chromosomal abnormalities in trisomy 18.

Our study has at least two limitations. First, due to the low availability of cord blood, only one healthy donor and one trisomy 18 fetal donor were available, which is hardly representative of the true characteristics of trisomy 18. At this stage, we can only characterize the common cell types. Our cell type assignments should be considered preliminary and ad hoc, and such assignments might vary considerably between independent studies. More detailed work will be necessary to efficiently dissect chromatin accessibility in rare cell types [37]. For example, further expansion of the sample size and sample type, or use cell models or animal models for extensive research and protocol improvement. The development of single-cell sequencing technology has made it easier for people to obtain gene and transcriptome information in a diverse organs and cell types at once. Recent research on fetal development has been greatly advanced [38–40], and it is enough to prove the huge prospects of single-cell sequencing technology in the field of development.

Nonetheless, our study reveals important molecules that may be associated with the development of trisomy 18: CCNB2 and MCM3 may be vital. These findings provide candidate molecules for future studies of trisomy 18. We found some differences in genes involved in pathways that may cause disease. For example, in human T cell leukemia virus 1 infection, although the specific mechanism by which the changes occur is unclear, the results suggest that the regulation of this pathway in trisomy 18 is altered. We also obtained several hundred genes that are differentially expressed from inaccessible chromatin regions of ME and progenitor. How these genes affect the occurrence and development of trisomy 18 is not yet known, but our findings will provide a molecular reference for future studies. It is worth further elucidating the specific role of these molecules in the development of trisomy 18 phenotypes.

## Conclusion

We have identified 6 cell populations in cord blood. Compared with the NC group, the ratio of B cells, progenitor cells, and megakaryocyte erythroid cells were significantly higher in the ES than the NC group. The ratios of natural killer (NK) cells, T cells, and monocyte/dendritic cell (ES) cells were significantly lower in the ES group than in the NC group. This result suggests that the immune system of trisomy 18 may have changed. Disorder in megakaryocyte erythroid cells implicates trisomy 18 in perturbing fetal hematopoiesis. We have found hundreds of differential peak genes in progenitor cells and megakaryocyte erythroid, while other cell populations have only 3 differential peak genes. The results suggest that progenitor cells and megakaryocyte erythroid cells may play a more important role in the occurrence and development of trisomy 18 than other cell populations. We identified a pathway in which the master differential regulatory pathway in the ME-0 cell population involves human T cell leukemia virus 1 infection, a pathway that is dysregulated in patients with trisomy 18 and which may increase the risk of leukemia in patients with trisomy 18. CCNB2 and MCM3 in progenitor may be vital to the development of trisomy 18. CCNB2 and MCM3, which have been reported to be essential components of the cell cycle and chromatin, may be related to chromosomal abnormalities in trisomy 18.

Our research has deepened our understanding of trisomy 18. At present, there are few studies on the mechanism of trisomy 18. The main limitation comes from improving the severity of the disease and the high mortality rate. The tissues include cell lines and animal models as well as 18-patients for research.

## Supplementary Information

**Additional file 1: Supplementary Figure 1.** (A) UMAP plots for the disease and control groups. (B) UMAP cluster to perform dimensionality reduction and clustering on the ES and NC data. (C) Expression of genes in the ES and NC groups in cluster 0 of megakaryocyte erythroid cells that participate in the human T cell leukemia virus 1 infection KEGG pathway. (D) Expression of genes in the ES and NC groups in cluster 1 of megakaryocyte erythroid cells that participate in the apoptosis KEGG pathway.

## Data Availability

The raw and processed data in this manuscript have been deposited with the Gene Expression Omnibus under accession number GSE158178. Related website is: https://www.ncbi.nlm.nih.gov/geo/query/acc.cgi?acc=GSE158178.
