# Supplementary material for: Single-cell chromatin accessibility landscape of human umbilical cord blood in trisomy 18 syndrome
Source: Hum Genomics. 2021 Jun 30;15:40. doi: 10.1186/s40246-021-00338-z (PMC8246660; doi:10.1186/s40246-021-00338-z)

Supplementary Material

**Supplementary Figure 1** (A) UMAP plots for the disease and control groups. (B) UMAP cluster to perform dimensionality reduction and clustering on the ES and NC data. (C) Expression of genes in the ES and NC groups in cluster 0 of megakaryocyte erythroid cells that participate in the human T-cell leukaemia virus 1 infection KEGG pathway. (D) Expression of genes in the ES and NC groups in cluster 1 of megakaryocyte erythroid cells that participate in the apoptosis KEGG pathway.


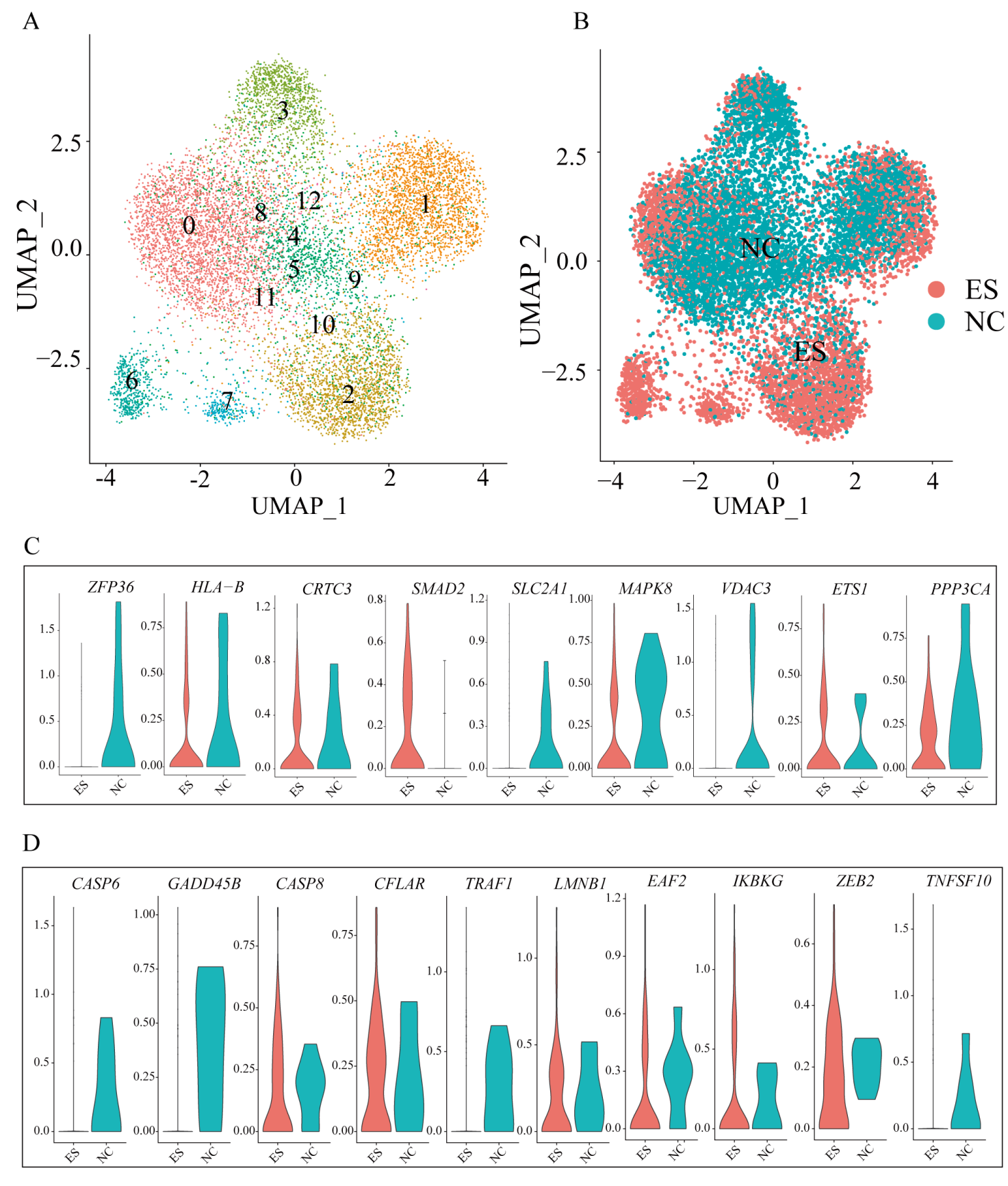

Supplement: Supplementary file 1 — Additional file 1: Supplementary Figure 1. (A) UMAP plots for the disease and control groups. (B) UMAP cluster to perform dimensionality reduction and clustering on the ES and NC data. (C) Expression of genes in the ES and NC groups in cluster 0 of megakaryocyte erythroid cells that participate in the human T cell leukemia virus 1 infection KEGG pathway. (D) Expression of genes in the ES and NC groups in cluster 1 of megakaryocyte erythroid cells that participate in the apoptosis KEGG pathway. [file 40246_2021_338_MOESM1_ESM.doc]
